# Benefits of colorectal cancer screening using fecal immunochemical testing with varying positivity thresholds by age and sex

**DOI:** 10.1093/jnci/djaf149

**Published:** 2025-06-23

**Authors:** Matthias Harlass, Amy B Knudsen, Daan Nieboer, Luuk A van Duuren, Karen M Kuntz, Carolyn M Rutter, Pedro Nascimento de Lima, Nicholson Collier, Jonathan Ozik, Anne I Hahn, Fernando Alarid-Escudero, Ann G Zauber, John M Inadomi, Reinier G S Meester, Iris Lansdorp-Vogelaar

**Affiliations:** Department of Public Health, Erasmus University Medical Center, Rotterdam, the Netherlands; Institute for Technology Assessment, Massachusetts General Hospital, Boston, MA, United States; Department of Radiology, Harvard Medical School, Boston, MA, United States; Department of Public Health, Erasmus University Medical Center, Rotterdam, the Netherlands; Department of Public Health, Erasmus University Medical Center, Rotterdam, the Netherlands; Division of Health Policy and Management, School of Public Health, University of Minnesota, Minneapolis, MN, United States; Biostatistics Program, Public Health Sciences Division, Hutchinson Institute for Cancer Outcomes Research, Fred Hutchinson Cancer Center, Seattle, WA, United States; Engineering and Applied Sciences Department, RAND Corporation, Arlington VA, United States; Decision and Infrastructure Sciences, Argonne National Laboratory, Lemont, IL, United States; Consortium for Advanced Science and Engineering, University of Chicago, Chicago, IL, United States; Decision and Infrastructure Sciences, Argonne National Laboratory, Lemont, IL, United States; Consortium for Advanced Science and Engineering, University of Chicago, Chicago, IL, United States; Department of Epidemiology and Biostatistics, Memorial Sloan Kettering Cancer Center, New York, NY, United States; Department of Health Policy, School of Medicine, Stanford University, Stanford, CA, United States; Stanford Health Policy, Freeman-Spogli Institute for International Studies, Stanford University, Stanford, CA, United States; Department of Epidemiology and Biostatistics, Memorial Sloan Kettering Cancer Center, New York, NY, United States; Department of Internal Medicine, The Spencer Fox Eccles School of Medicine, University of Utah, Salt Lake City, UT, United States; Department of Public Health, Erasmus University Medical Center, Rotterdam, the Netherlands; Health Economics and Outcomes Research, Freenome Holdings Inc, South San Francisco, CA, United States; Division of Gastroenterology and Hepatology, School of Medicine, Stanford University, Stanford, CA, United States; Department of Public Health, Erasmus University Medical Center, Rotterdam, the Netherlands

## Abstract

**Background:**

Fecal immunochemical test (FIT) performance for colorectal cancer screening varies by age and sex, yet most FIT-based screening programs use uniform positivity thresholds. This study assessed the potential benefits of stratifying FIT thresholds based on age and sex.

**Methods:**

We conducted a meta-analysis of FIT sensitivity and specificity at various positivity thresholds by age and sex. We then used these estimates in 2 microsimulation models of colorectal cancer and projected lifetime clinical outcomes, incremental costs, and quality-adjusted life-years (QALYs) gained from age- and sex-stratified FIT strategies. FIT thresholds ranged from 10 to 50 µg hemoglobin per gram of feces.

**Results:**

For current uniform FIT screening (20 µg hemoglobin/gram of feces), models projected 85.67 to 122.15 QALYs gained at incremental costs of ‒$982 to $504 per 1000 individuals compared with no screening. At equivalent costs to current uniform screening, only 1 model found stratified FIT approaches cost-effective, yielding a marginal increase of 1.04 and 1.10 QALYs gained/1000 female and male individuals, respectively. At a willingness-to-pay threshold of $100 000/QALYs gained, both models found stratified FIT cutoffs to be the best strategy, with cutoffs being equal to or higher for males and lowest at older ages (70-75 years). Uniform strategies showed comparable effectiveness, falling within 1 quality-adjusted life-day per person of efficient strategies at up to $112 more per person. Results were sensitive to FIT test performance characteristics and 1-time setup costs.

**Conclusion:**

Stratifying FIT thresholds by age and sex may be cost-effective compared to current screening. The gain in expected health benefits with stratified FIT screening, however, is likely small.

## Introduction

The United States Preventive Services Task Force (USPSTF) recommends annual fecal immunochemical tests (FITs) for individuals aged 45-75 years as an effective method of colorectal cancer (CRC) screening.^1^ A FIT is a quantitative test, and decision makers can set the hemoglobin concentration above which a test is positive. Most FIT-based screening programs use uniform positivity thresholds, despite males having a higher CRC risk than females.^2^ Females with screen-detected CRC exhibit lower fecal hemoglobin levels, indicating reduced test sensitivity at equivalent thresholds.^3-7^ Moreover, multiple studies have reported lower CRC mortality reductions in females from screening.^8-10^

To achieve equity in screening benefits, Finnish regions implemented sex-based FIT screening, and Swedish regions implemented gender-based FIT screening, applying lower cutoffs for females and women, respectively.^11-13^ After 2 screening rounds using 40 µg hemoglobin per gram of feces in women and 80 µg hemoglobin per gram of feces cutoffs in men, the Swedish program achieved comparable interval cancer rates across genders.^14^ Finnish first-year results demonstrated improved CRC detection rates, particularly in females, compared with previous uniform guaiac fecal occult blood test screening.^12^ Subsequent modeling of a Finnish population showed that optimal starting and stopping ages and positivity thresholds differed by sex, suggesting broader applicability of sex-stratified FIT screening approaches.^15^

In addition to sex-specific differences, there is compelling evidence that fecal hemoglobin concentrations and FIT performance change with age.^6^^,^^16^^,^^17^ A meta-analysis revealed that OC-Sensor FIT (Eiken Chemical Co) sensitivity at 20 µg hemoglobin per gram of feces declined from 85% at ages 50-59 years to 73% at ages 60-69 years.^6^ Moreover, CRC risk increases with age.^2^ Using lower cutoffs at older ages may counterbalance observed age-related declines in sensitivity, although at the expense of reduced specificity. Despite prior studies suggesting benefits from age- and sex-specific thresholds, no previous study has comprehensively assessed this approach.

Our primary objective was to evaluate the screening burden, benefits, and cost-effectiveness of FIT screening for CRC with varying positivity thresholds based on age and sex compared with screening with uniform thresholds.

## Methods

We first conducted a meta-analysis of published FIT performance characteristics to estimate sensitivity and specificity for CRC and adenomas at various positivity thresholds by age and sex. Subsequently, we used these estimates in 2 independently developed microsimulation models (Microsimulation Screening Analysis–Colon [MISCAN-Colon] and Simulation Model of Colorectal Cancer [SimCRC]).

### Meta-analysis of FIT test characteristics

We performed a meta-analysis to estimate FIT sensitivity for detecting CRC, advanced adenomas, and nonadvanced adenomas as well as specificity for having no neoplasms, simultaneously stratified by age, sex, and positivity threshold. A detailed method description is available in Supplementary Materials. In short, we used a meta-analysis method that models sensitivity and specificity as functions of the positivity threshold, allowing interpolation and extrapolation to relevant thresholds for this analysis.^18^ Given limited published data on test characteristics, we incorporated assumptions regarding sex-specific performance variations across age strata. Test characteristics for the primary analysis are presented in Table 1.

**Table 1.** FIT performance characteristics, by age, sex, and positivity threshold, used in the analysis

Sex	Age, y	Positivity threshold, µg hemoglobin per g of feces	Sensitivity					Specificity
			CRC	Adenoma				
				≥10 mm		6 to <10 mm	1 to <6 mm	
**FIT**								
Female	45-59	10	0.835	0.337	0.102		0.091	0.909
		20	0.757	0.265	0.067		0.051	0.949
		30	0.701	0.232	0.056		0.037	0.963
		40	0.657	0.212	0.050		0.029	0.971
		50	0.621	0.199	0.047		0.024	0.976
	60-69	10	0.794	0.324	0.107		0.103	0.897
		20	0.718	0.253	0.066		0.056	0.944
		30	0.666	0.222	0.054		0.039	0.961
		40	0.627	0.204	0.048		0.030	0.970
		50	0.595	0.192	0.045		0.025	0.975
	70-75	10	0.770	0.318	0.118		0.117	0.883
		20	0.697	0.247	0.068		0.064	0.936
		30	0.649	0.217	0.053		0.045	0.955
		40	0.612	0.199	0.046		0.034	0.966
		50	0.583	0.188	0.043		0.028	0.972
Male	45-59	10	0.863	0.350	0.109		0.105	0.895
		20	0.786	0.276	0.069		0.061	0.939
		30	0.727	0.241	0.055		0.043	0.957
		40	0.680	0.220	0.049		0.034	0.966
		50	0.640	0.206	0.045		0.028	0.972
	60-69	10	0.832	0.340	0.120		0.120	0.880
		20	0.753	0.266	0.071		0.067	0.933
		30	0.697	0.232	0.055		0.047	0.953
		40	0.653	0.212	0.047		0.037	0.963
		50	0.617	0.199	0.043		0.030	0.970
	70-75	10	0.800	0.331	0.138		0.138	0.862
		20	0.723	0.258	0.078		0.078	0.922
		30	0.671	0.226	0.057		0.055	0.945
		40	0.631	0.207	0.047		0.043	0.957
		50	0.598	0.194	0.042		0.035	0.965
**Colonoscopy**								
All	All	—	0.950	0.950	0.850		0.750	0.860^a^

Abbreviations: CRC = colorectal cancer; FIT = fecal immunochemical test.

a The lack of specificity for colonoscopy reflects the detection and removal of nonadenomatous polyps.

### Model descriptions

We used 2 independently developed microsimulation models of CRC (MISCAN-Colon and SimCRC) that are part of the Cancer Intervention and Surveillance Modeling Network (CISNET) and previously informed USPSTF screening recommendations.^1^ Extensive model descriptions are published elsewhere.^19-21^

**Figure 1.**
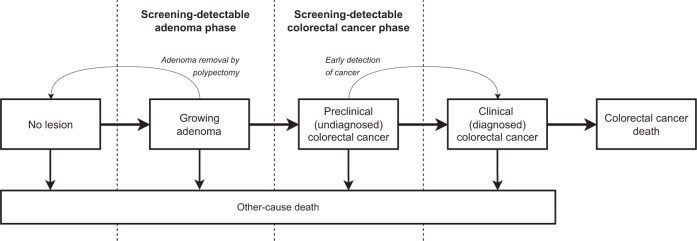
Colorectal cancer natural history and screening effects simulated by the Microsimulation Screening Analysis–Colon Model and the Simulation Model of Colorectal Cancer.

Briefly, both models simulate individual life histories in a large cohort that represents US population distributions for life expectancy and CRC risk. The models simulate CRC natural history and screening impacts (Figure 1). We used identical model parameters and assumptions as a previous modeling analysis, incorporating recent trends in CRC incidence.^1^

### Simulated screening strategies

We simulated annual FIT screening from ages 45 to 75 years with varying positivity thresholds in cohorts of 40-year-old females and males previously unscreened and free from clinical CRC. We divided the recommended age range for annual FIT screening into 3 groups (45-59, 60-69, and 70-75 years) and varied positivity thresholds between 10 μg hemoglobin per gram of feces and 50 μg hemoglobin per gram of feces in increments of 10 μg hemoglobin per gram of feces per group. Strategies were labeled “FIT-X/Y/Z,” where X, Y, and Z represent thresholds in μg hemoglobin per gram of feces for ages 45-59, 60-70, and 70-75 years, respectively. This approach generated 125 unique FIT strategies (Table S1). Individuals with hemoglobin concentrations above the positivity threshold underwent follow-up colonoscopy. Individuals with adenomas detected at follow-up colonoscopy entered surveillance according to published guidelines (Table S2).^22^ We assumed 100% adherence to screening, follow-up, and surveillance. Colonoscopy parameters and complication rates are documented in Table S3.

### Costs and disutilities

Costs and disutility assumptions are presented in Tables S4 to S7. Costs were calculated from a modified societal perspective, encompassing direct medical and patient time costs but excluding direct nonmedical and broader societal spillover effects. Costs for FIT, screening-related complications, and cancer care were updated from Peterse et al.^23^ to 2020 US dollars using the Consumer Price Index.^24^ Colonoscopy costs were calculated using 2020 Centers for Medicare & Medicaid Services data. To calculate quality-adjusted life-years (QALYs), we incorporated disutilities—decrements in health-related quality of life—in line with previous analyses.^23^ Disutilities captured the impact of screening procedures, including anxiety during waiting periods and cancer-related quality of life decrements. Future costs and QALYs were discounted at 3% annually.

### Projected outcomes

We calculated the following lifetime outcomes per 1000 individuals for all screening strategies: CRC cases, CRC deaths, colonoscopies, complications, life-years, QALYs, and total costs. For each sex and model, we identified efficient screening strategies using cost-effectiveness analysis. A strategy was efficient if no other strategy (or combination of strategies) achieved better health outcomes (QALYs) at equivalent or lower cost. We plotted the efficient frontier connecting efficient strategies and calculated incremental cost-effectiveness ratios (ICERs) between adjacent efficient options. ICERs were estimated by dividing additional costs by QALYs gained and rounded to the nearest $100. The optimal strategy was defined as the most effective strategy with an ICER below the willingness-to-pay threshold of $100 000/QALYs gained.

### Sensitivity analyses

#### Probabilistic sensitivity analysis

We conducted a probabilistic sensitivity analysis to evaluate the impact of uncertainty in FIT sensitivity and specificity estimates (ie, parameter uncertainty) and assess their influence on model outcomes. From 2 independent posterior distributions estimated through meta-analysis, we sampled 100 functions relating threshold to sensitivity and specificity for CRC, stratified by age and sex and unstratified for advanced adenoma. For advanced adenoma, we applied age- and sex-specific scaling factors derived from the CRC model to generate stratified estimates, whereas nonadvanced adenoma parameters were derived using fixed scaling ratios from advanced adenoma estimates. Sensitivities for different disease stages (CRC, advanced adenoma, nonadvanced adenoma) were combined and correlated using an algorithm described in the Supplementary Materials. 

#### Value of information analysis

We conducted a value of information analysis using expected loss curves to quantify the clinical and economic implications of potentially choosing suboptimal screening strategies due to uncertainty in FIT performance. Expected loss curves quantify the loss in net monetary benefit of choosing a suboptimal strategy and the expected value of perfect information, a measure that indicates how much value could be gained by eliminating all uncertainty in FIT performance characteristics.^25^^,^^26^ To contextualize potential population-level implications, we scaled lifetime per-person estimates to a single US birth cohort reaching screening eligibility in 2020 (approximately 4 million eligible individuals).^27^^,^^28^ Detailed methodology for the value of information analysis is described in Supplementary Materials.

#### Costs for nonuniform positivity thresholds

Stratifying FIT thresholds by age and sex may induce extra costs due to the increased administrative burdens of stratification. We evaluated the impact of 1-time additional costs at 50%, 100%, and 200% of a single FIT ($44) for all individuals beginning screening at age 45 years.

#### Reduced adherence to screening and surveillance

Our primary analysis assumed perfect adherence to FIT screening and colonoscopies, enabling us to assess potential benefits for guideline-adherent individuals. Actual adherence is often much lower, however, altering the effectiveness of different screening strategies. To address this discrepancy, we conducted 2 sensitivity analyses on adherence assumptions. In the first reduced-adherence scenario, we simulated strategies assuming 60% adherence to FIT screening, diagnostic colonoscopies, and surveillance colonoscopies. We treated adherence to each test as independent. After entering surveillance, nonadherent individuals would not reenter screening but be invited for their next surveillance colonoscopy after the same interval as the missed appointment.

In the second reduced-adherence scenario, we assumed that 10% of individuals would never attend screening and 30% would always participate. The remaining population has a 60% probability of attending the first screening test. After the initial test, individuals who attended have a 75% chance of attending the next screening, whereas nonattenders have a 25% chance of attending the next screening. The diagnostic and surveillance colonoscopy assumptions were identical to the first adherence sensitivity analysis.

#### Biennial and triennial screening

The optimal threshold combinations may vary under longer screening intervals, which are frequently used in settings outside the United States. We evaluated all strategies from our primary analysis under biennial and triennial screening intervals, assuming perfect adherence to all tests.

## Results

### Screening with the current uniform FIT threshold vs no screening

Screening with the current uniform FIT threshold of 20 µg hemoglobin per gram of feces was projected to lead to a considerable gain in QALYs, averted CRC cases, and averted CRC deaths compared with no screening. Over the lifetimes of 1000 females, the models projected 86-105 QALYs gained, 38-54 CRC cases averted, and 23-26 CRC deaths averted while requiring 1779-1943 additional colonoscopies and incremental costs of ‒$606 to $504 per individual (range is across models; see Table S8). Males exhibited greater projected benefits (96-122 QALYs gained, 42-66 CRC cases averted, 26-32 CRC deaths averted per 1000 men), more colonoscopies (2019-2176 per 1000 males), and higher incremental costs (‒$982 to $476 per person). Note that a negative incremental cost implies that the strategy is cost saving, meaning that it yields the respective health outcomes at a lower cost than the comparator strategy.

### Screening with age- and sex-specific FIT thresholds vs the current uniform threshold

**Figure 2.**
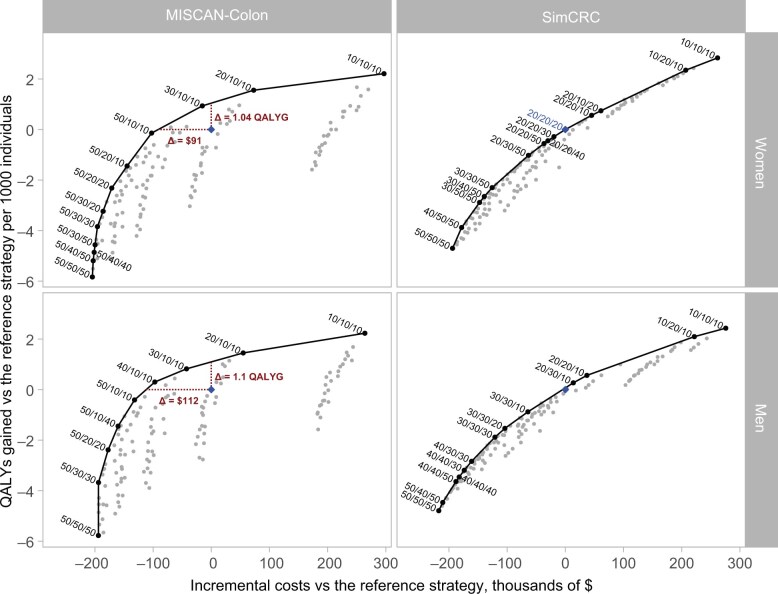
Costs and QALYs gained for all FIT threshold combinations, by model and sex. Strategies are labeled “X/Y/Z,” where X, Y, and Z represent thresholds in μg hemoglobin per gram of feces for ages 45-59 years, 60-70 years, and 70-75 years, respectively. The blue diamond marks the current uniform FIT threshold of 20 µg hemoglobin per gram of feces, which is the reference strategy. FIT = fecal immunochemical test; MISCAN-Colon = Microsimulation Screening Analysis–Colon; QALY = quality-adjusted life-year; SimCRC = Simulation Model of Colorectal Cancer.

The other evaluated threshold combinations yielded varying benefits, screening burdens, and cost outcomes vs current FIT screening (Figure 2). Higher cutoffs consistently reduced QALYs gained, colonoscopies, and costs across both models, whereas a lower cutoff of 10 µg hemoglobin per gram of feces had the opposite effect. For example, with MISCAN-Colon, QALYs gained compared with current uniform screening ranged from ‒6 to 2 per 1000 females at uniform thresholds of 50 and 10 µg hemoglobin per gram of feces, respectively (Table S9), incremental colonoscopies ranged from ‒611 to 545 per 1000 individuals, and incremental costs between ‒$204 and $297 per individual. Both models showed lower ranges for males vs females, with SimCRC demonstrating slightly higher QALYs gained ranges and incremental colonoscopies than MISCAN-Colon for males and females (Table S9).

### Efficient strategies

The specific threshold combinations on the efficient frontier varied depending on the model and sex (Figure 2; Table 2). In the MISCAN-Colon model, when accepting higher costs compared with the current uniform screening, the most efficient approach for males and females was first to lower the FIT cutoffs for the age groups 60-69 years and 70-75 years. Once these age groups reached the lowest cutoff of 10 µg hemoglobin per gram of feces, the cutoff for the 45-59 years age group was then decreased. In contrast, for females in SimCRC, the cutoffs for the age groups 45-59 years and 60-69 years tended to decrease first as costs increased, whereas no such pattern was observed for males (Figure 2; Table 2).

**Table 2.** Outcomes per 1000 40-year-olds for no screening, current FIT screening, and efficient FIT screening strategies

Model	Sex	Threshold, by age in µg hemoglobin per g of feces^a^	Outcomes per 1000 40-y-olds					Incremental cost-effectiveness ratio
			CRC cases, No.	CRC deaths, No.	Colonoscopies, No.	QALYs	Costs, thousands of $	
MISCAN-Colon	Female	No screening	78.1	33.2	78	19 924	4566	—
		20/20/20^b^	40.4	10.0	2021	20 010	5070	—
		50/50/50	47.0	11.7	1410	20 004	4865	(Referent)
		50/40/50	46.4	11.6	1450	20 005	4867	2200
		50/40/40	46.0	11.4	1474	20 005	4868	4900
		50/30/50	45.5	11.3	1511	20 005	4870	5300
		50/30/30	44.4	10.9	1569	20 006	4874	5700
		50/30/20	43.5	10.6	1632	20 007	4884	15 900
		50/20/20	42.3	10.3	1726	20 007	4899	16 300
		50/20/10	40.5	9.7	1852	20 008	4925	30 200
		50/10/10	38.8	9.4	2022	20 010	4967	32 300
		30/10/10	38.1	9.3	2156	20 011	5054	81 000
		20/10/10	37.5	9.3	2291	20 011	5143	142 100
		10/10/10	36.5	9.2	2566	20 012	5367	342 500
	Male	No screening	87.1	36.2	87	19 276	5211	—
		20/20/20^b^	45.2	10.5	2264	19 372	5687	—
		50/50/50	52.6	12.1	1635	19 366	5493	(Referent)
		50/30/30	49.8	11.3	1797	19 368	5493	100
		50/20/20	47.4	10.8	1943	19 369	5510	12 800
		50/10/40	46.0	10.7	2058	19 370	5527	18 200
		50/10/10	44.1	10.0	2200	19 371	5555	27 300
		40/10/10	43.6	10.0	2260	19 372	5590	49 100
		30/10/10	43.2	9.9	2348	19 373	5645	104 500
		20/10/10	42.5	9.9	2490	19 373	5742	154 800
		10/10/10	41.2	9.9	2762	19 374	5951	267 900
SimCRC	Female	No screening	78.5	31.5	78	19 922	4674	**—**
		50/50/50	30.2	6.8	1294	20 023	3874	(Referent)
		40/50/50	29.6	6.6	1352	20 024	3889	19 000
		30/50/50	28.7	6.5	1438	20 024	3920	31 000
		30/40/50	28.3	6.4	1471	20 025	3928	34 700
		30/30/50	27.8	6.2	1523	20 025	3942	38 400
		20/30/50	26.5	6.0	1666	20 026	4004	48 900
		20/20/50	25.6	5.8	1752	20 027	4031	57 100
		20/20/40	25.4	5.7	1771	20 027	4038	60 900
		20/20/30	25.1	5.6	1802	20 027	4048	63 800
		20/20/20^b^	24.5	5.4	1857	20 027	4068	69 500
		20/20/10	23.1	5.0	1985	20 028	4113	80 300
		20/10/20	22.8	5.1	2027	20 028	4129	88 700
		10/20/10	21.1	4.7	2269	20 030	4275	90 700
		10/10/10	20.0	4.5	2403	20 030	4329	112 500
	Male	No screening	91.9	37.6	92	19 272	5370	—
		20/20/20^b^	26.0	5.8	2111	19 395	4388	—
		50/50/50	32.0	7.3	1528	19 390	4171	(Referent)
		50/40/50	31.5	7.2	1565	19 390	4177	20 900
		40/40/50	30.9	7.0	1629	19 391	4200	27 400
		40/40/40	30.6	6.9	1651	19 391	4206	30 900
		40/40/30	30.1	6.7	1686	19 391	4214	32 700
		40/30/30	29.4	6.6	1738	19 392	4227	35 500
		30/30/30	28.6	6.4	1829	19 393	4267	42 000
		30/30/20	27.9	6.2	1887	19 393	4284	49 000
		30/30/10	26.3	5.7	2009	19 394	4324	60 500
		20/30/10	25.3	5.5	2156	19 395	4402	67 900
		20/20/10	24.6	5.4	2221	19 395	4425	79 400
		10/20/10	23.0	5.2	2501	19 397	4610	120 400
		10/10/10	22.1	5.1	2621	19 397	4664	161 100

Abbreviations: CRC = colorectal cancer; FIT = fecal immunochemical test; MISCAN-Colon = Microsimulation Screening Analysis–Colon; QALY = quality-adjusted life-year; SimCRC = Simulation Model of Colorectal Cancer.

a Strategies were denoted as “FIT-X/Y/Z,” where X, Y, and Z are thresholds in μg hemoglobin per gram of feces for individuals aged 45-59 years, 60-69 years, and 70-75 years, respectively.

b Strategy FIT-20/20/20 is the current FIT screening strategy in the United States using OC-Sensor FIT.

The current uniform FIT screening strategy (at 20 µg hemoglobin per gram of feces) was on or near the efficient frontier in SimCRC but not for MISCAN-Colon. For MISCAN-Colon, current uniform screening among females and males provided marginally fewer QALYs gained and was slightly more costly than other strategies. At equivalent costs to current uniform screening, only MISCAN-Colon found age- and sex-specific FIT approaches cost-effective, yielding a marginal increase of 1.04 and 1.10 QALYs gained per 1000 females and males, respectively (Figure 2). This approach alternatively reduced per-person screening costs by $94 (females) and $112 (males) while maintaining health benefits in MISCAN-Colon.

### Cost-effectiveness of uniform vs nonuniform FIT thresholds

Strategies with uniform thresholds of 10 and 50 µg hemoglobin per gram of feces were anticipated to land on the lower and upper end of the efficient frontier, respectively, being the most and least intensive options regarding follow-up colonoscopies, the main contributor to screening costs. SimCRC was the only model that found a uniform FIT threshold other than 10 and 50 µg hemoglobin per gram of feces to be an efficient option—namely, FIT-20/20/20 in females and FIT-40/40/40 and FIT-30/30/30 in males (strategies are denoted as “FIT-X/Y/Z,” where X, Y, and Z are thresholds in μg hemoglobin per gram of feces for individuals aged 45-59 years, 60-69 years, and 70-75 years, respectively). All uniform strategies, however, were on or within 1 quality-adjusted life-day per person of the efficient frontier in both models, indicating that they were either equally effective or marginally inferior compared with (combinations of) age- and sex-stratified strategies.

With MISCAN-Colon, FIT-30/10/10 was optimal for females, with an ICER of $81 000 per QALY gained, whereas FIT-40/10/10 was optimal for males, with an ICER of $49 100 per QALY gained (Table 2). Although FIT-30/10/10 was also efficient for males, its ICER ($104 500 per QALY gained) slightly exceeded the willingness-to-pay threshold. Compared with uniform screening at 20 µg hemoglobin per gram of feces, the optimal stratified strategies yielded distinct outcomes by sex. Males had fewer CRC cases averted (1.6 vs 2.5) and CRC deaths averted (0.5 vs 0.6) per 1000 individuals than females did (Table 2), although males required 3 fewer colonoscopies than uniform screening, whereas females needed 135 additional colonoscopies per 1000 individuals. Males also achieved greater cost savings per person ($97 vs $15), despite lower QALYs gained (0.3 vs 0.9 per 1000 individuals) (Table 2).

With SimCRC, FIT-10/20/10 and FIT-20/20/10 were the optimal strategies for females and males, with ICERs of $90 700 and $79 400 per QALY gained, respectively (Table 2). The optimal strategies showed similar sex-specific patterns to MISCAN-Colon: Compared with current uniform screening, males had fewer CRC cases averted (21.1 vs 24.6) and CRC deaths averted (4.7 vs 5.4) per 1000 individuals than females did. Males required substantially fewer additional colonoscopies (110 vs 412 per 1000 individuals) and incurred lower incremental costs per person ($37 vs $207) while having fewer QALYs gained (0.6 vs 2.4 per 1000 individuals).

### Sensitivity analyses

#### Probabilistic sensitivity analysis

**Figure 3.**
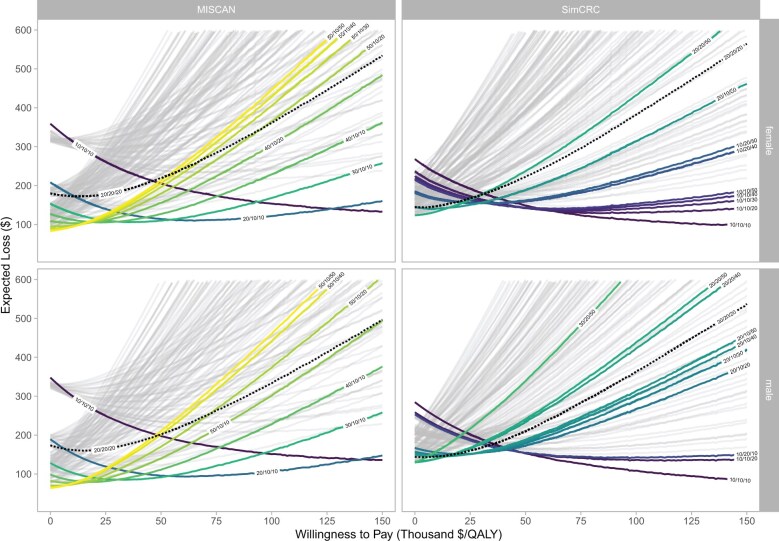
Expected loss per individual for all FIT threshold combinations. Strategies are labeled “X/Y/Z,” where X, Y, and Z represent thresholds in μg hemoglobin per gram of feces for ages 45-59 years, 60-70 years, and 70-75 years, respectively. The colored lines represent strategies that minimize the expected loss for some value of the willingness-to-pay threshold. The black dotted line represents the current uniform threshold of 20 µg hemoglobin per gram of feces. FIT = fecal immunochemical test; MISCAN-Colon = Microsimulation Screening Analysis–Colon; QALY = quality-adjusted life-year; SimCRC = Simulation Model of Colorectal Cancer.

The probabilistic sensitivity analysis showed that the strategies with the lowest expected loss at a willingness-to-pay threshold of $100 000 per QALY gained were more intensive than current FIT screening—namely, FIT-20/10/10 in MISCAN-Colon and FIT-10/10/10 in SimCRC for both sexes (Figure 3). The difference in net monetary benefit between current and optimal strategies was $227-$271 per person. The expected value of perfect information for a single birth cohort, assuming 83% of 45-year-olds were screening eligible in 2020, ranged from $435 million to $450 million.

#### Additional costs for nonuniform positivity thresholds

One-time implementation costs (50%, 100%, or 200% of a single FIT) applied at age 45 years substantially affected strategy efficiency and cost-effectiveness (Figure S1). In SimCRC, all uniform strategies were efficient across all implementation cost scenarios. MISCAN-Colon showed that uniform screening at 30 and 40 µg hemoglobin per gram of feces became efficient only when implementation costs reached 200% of a single FIT, whereas 20 µg hemoglobin per gram of feces remained dominated (Figure S1). Probabilistic sensitivity analysis revealed that SimCRC’s optimal strategy remained FIT-10/10/10 at $100 000 per QALY gained willingness to pay (Figure S2). MISCAN-Colon favored FIT-20/10/10 in most scenarios, except for females at 200% implementation costs, where FIT-10/10/10 was optimal.

#### Reduced adherence to screening and surveillance

Lowering adherence to screening, follow-up, and surveillance decreased screening effectiveness and ICERs across all strategies. The relative reduction in screening effectiveness and colonoscopy use was comparable between males and females. At a fixed 60% adherence rate, current uniform screening resulted in 41%-85% more cancer cases, 69%-128% more cancer deaths, and 25%-32% fewer QALYs gained while requiring 49%-52% fewer colonoscopies compared with a scenario with perfect adherence (range across models and sexes). In the second variable-adherence scenario, the number of cancer cases and deaths under current screening increased by 37%-68% and 77%-116%, respectively. The QALYs gained were 23%-35% lower, whereas colonoscopies decreased by 37%-43%.

In both scenarios, efficient strategies shifted toward lower thresholds, whereas differences between the models reflected the patterns observed in the primary analysis: MISCAN-Colon favored higher thresholds at ages 45-60 years and lower thresholds for ages 60-75 years, whereas SimCRC tended to have lower thresholds up to age 69 years and high thresholds for ages 70-75 years (Figures S3 and S4). Moreover, the benefits of stratified FIT screening compared with current screening were greater in scenarios with reduced adherence than under perfect adherence. In MISCAN-Colon, efficient strategies favored higher thresholds for ages 45-60 years but lower thresholds for ages 60-75 years. Conversely, efficient strategies in SimCRC tended to have lower thresholds up to age 69 years and high thresholds for ages 70-75 years (Figures S3 and S4). In both scenarios with reduced adherence, none of the efficient strategies exceeded the willingness-to-pay threshold of $100 000 per QALY gained. Thus, the strategy with the lowest, most sensitive positivity threshold (10/10/10), which offered the greatest health benefit, was considered cost-effective.

#### Biennial and triennial screening

Longer screening intervals were associated with a shift of optimal strategies toward lower, more sensitive positivity thresholds (Figures S5 and S6). In MISCAN-Colon, most efficient biennial or triennial FIT strategies had 10 µg hemoglobin per gram of feces thresholds for ages 60-75 years. For SimCRC, efficient biennial or triennial strategies had lower thresholds until 69 years of age and varied thresholds for ages 70-75 years.

## Discussion

At costs equivalent to current uniform screening (20 µg hemoglobin per gram of feces), age- and sex-stratified FIT was cost-effective only in the MISCAN-Colon model. When considering higher-cost strategies within the willingness-to-pay threshold of $100 000 per QALY gained, stratified positivity thresholds yielded additional QALYs gained over current screening in both models. In the base-case analysis, cost-effective strategies used lower thresholds than current practice, except in the youngest age group in MISCAN-Colon. Cost-effective strategies were FIT-30/10/10 or FIT-10/20/10 for females and FIT-40/10/10 or FIT-20/20/10 for males in MISCAN-Colon and SimCRC, respectively. Given FIT performance uncertainty, even lower thresholds (FIT-20/10/10 in MISCAN-Colon; FIT-10/10/10 in SimCRC) maximized the expected net monetary benefit at $100 000 per QALY gained for both sexes in our probabilistic sensitivity analysis. Sensitivity analyses indicated that high implementation costs for stratified thresholds generally favor uniform FIT cutoffs.

Expected health benefits from stratified FIT screening were modest, potentially because of 2 key factors. First, stratification was limited to positivity thresholds within an annual screening strategy without modifying intervals or modalities, which may have a greater impact on screening outcomes. Second, because of a lack of data on FIT characteristics below 10 μg hemoglobin per gram of feces, we did not evaluate lower thresholds. Many efficient combinations partially used the current threshold (20 μg hemoglobin per gram of feces) or the lowest evaluated threshold (10 μg hemoglobin per gram of feces). It remains uncertain whether even lower thresholds would produce more favorable outcomes.

Both models identified lower optimal thresholds for females than for males, but they varied in projected health outcomes, costs, and efficient strategies, likely because of differences in model structures. Although both models were calibrated to the same observed adenoma prevalence and cancer incidence data, a previous systematic comparison revealed important differences in their underlying assumptions.^29^ The MISCAN-Colon model assumes that adenomas that develop earlier in life have a lower probability of progressing to cancer than adenomas that develop later in life, whereas the SimCRC model assumes that age at adenoma development is not tied to the probability of progression to cancer. This difference likely explains why the MISCAN-Colon model favors higher thresholds for younger age groups, whereas the SimCRC model favors more uniform thresholds across age groups.

The optimal strategies identified through probabilistic sensitivity analysis differ from the deterministic findings in our primary analysis and warrant nuanced interpretation for 2 reasons. First, health technology assessment guidelines often recommend probabilistic analyses for nonlinear models to avoid bias in mean outcome estimates.^30^ These analyses, however, can paradoxically introduce greater bias when parameter distributions exhibit high skewness and large variances, as observed in our FIT sensitivity and specificity parameters.^31^ Moreover, the computational demands of the models prohibit large probabilistic sensitivity analysis samples, which would improve the accuracy of estimated expected values.^32^ Given these constraints, we used deterministic estimates in our primary analysis. Second, expected loss curves identify strategies that minimize average loss (maximize average net monetary benefit), which may yield different recommendations than those derived from deterministic analyses using ICERs.^25^ Risk-averse policymakers may prefer strategies with lower expected losses over those demonstrating superior cost-effectiveness in deterministic analyses. Although the expected monetary value per person of eliminating all uncertainty in FIT characteristics in our value of information analysis was small, the potential value of further research may be considerable, given the large screening-eligible population, reflected in the estimated expected value of perfect information of $435 million to $450 million for a birth cohort, screening eligible in 2020.

Adherence is a key assumption in decision analytic cancer screening models^33^ and a strong determinant of screening effectiveness, as observed in our sensitivity analyses. Our analysis showed that reduced adherence leads to longer screening intervals and diminished effectiveness. The cost-effectiveness improves, however, due to the diminishing returns of frequent screening in an annual FIT strategy. This behavior also becomes apparent from the lowest positivity threshold being cost-effective for all ages and sexes in reduced-adherence scenarios. Nonetheless, population-level recommendations of optimal strategies should not be based on analyses under nonadherence. Although real-world screening adherence is imperfect, basing optimal strategies on imperfect adherence assumptions can lead to more intensive recommendations,^34^ potentially resulting in overscreening individuals who follow guidelines. Pedersen et al.^34^ illustrated this concept with an analogy: Recommending paracetamol (acetaminophen) every 4 hours instead of every 8 hours to account for forgetful individuals could lead to potentially harmful outcomes in individuals who follow the prescription. Similarly, “optimal” screening strategies identified in scenarios with reduced adherence may inadvertently lead to harmful recommendations for individuals who are adherent. One important exception is adaptive strategies, which adjust screening recommendations to an individual’s recent screening behavior. Previous analyses have shown notable variations in expected health outcomes and cost-effectiveness of screening based on screening history, demonstrating the potential value of more complex, risk-adaptive screening strategies.^35^

The scope of our analysis, however, was the stratification of FIT screening by age and sex at the population level. Therefore, our primary analysis assumed 100% adherence to identify optimal strategies based on cost-effectiveness. The goal of our sensitivity analyses was to demonstrate the expected outcomes of different strategies under conditions of suboptimal adherence rather than to use these scenarios to determine optimal screening strategies applicable in real-world situations. Overall, we observed that a lack of adherence increases differences in health outcomes and cost-effectiveness between stratified uniform strategies.

Previous research on sex-specific FIT thresholds is limited, particularly regarding lifetime outcomes and the cost-effectiveness of stratification. A Dutch study evaluated the cost-effectiveness of gender-specific FIT strategies and varied positivity thresholds, starting ages, stopping ages, and screening intervals.^36^ The study’s findings indicated that gender-specific screening was preferable at lower willingness-to-pay thresholds, though uniform screening achieved comparable benefits at a higher willingness to pay (more than €20 000/QALY gained). The results of a Finnish pilot trial indicated that sex-specific FIT thresholds (70 µg hemoglobin per gram of feces for males and 25 µg hemoglobin per gram of feces for females) led to greater improvements in outcomes in females over males from the previously used guaiac fecal occult blood test. Subsequent simulation modeling of the cost-effectiveness of sex-specific FIT screening in Finland using MISCAN-Colon identified optimal strategies of 10 µg hemoglobin per gram of feces for females aged 55-69 years and 50 µg hemoglobin per gram of feces for males aged 50-79 years, aligning with our finding that females benefit from lower thresholds.^15^

Implementing stratified FIT cutoffs presents multiple challenges. Changes to existing FIT programs could affect screening adherence, a critical determinant of screening effectiveness. With tests such as FIT, suboptimal adherence to initial screening and potential follow-up colonoscopies must be considered. Second, lowering positivity thresholds increases follow-up colonoscopy demand, potentially straining resource-limited settings. The optimal strategies in our study used lower thresholds for females and at older ages than current screening, requiring about 21% more colonoscopies in total. Long waiting times from positive FIT result to follow-up colonoscopy would result in worse health outcomes than reported in our analysis.^37^ However, FIT screening at low thresholds still requires fewer colonoscopies than primary colonoscopy screening, potentially benefiting resource-limited settings.^1^^,^^38^ Our study did not evaluate thresholds below 10 µg hemoglobin per gram of feces, which could increase sensitivity but also increase false positives.^6^ Further decreasing positivity thresholds may also yield inconsistent results when encountering the lower analytical range limitations of current testing platforms.

Our study has important limitations. First, our estimation approach for age- and sex-specific FIT characteristics assumes consistency in relative sex-based performance differentials across age cohorts. Second, because of a lack of data, we had to make several assumptions to derive FIT sensitivity by adenoma size. Third, uncertainty in FIT characteristics varied by positivity threshold, with greater uncertainty for thresholds below 20 μg hemoglobin per gram of feces due to limited empirical data, whereas higher thresholds had more robust evidence and consequently more precise pooled sensitivity estimates. Fourth, our probabilistic sensitivity analysis on FIT characteristics was constrained to 100 parameter sets because of the substantial computational burden involved. A larger number of parameter sets may have captured a wider range of parameter uncertainty. Fifth, we did not consider individual variations in cancer risk beyond the stratification by age and sex. Future research could evaluate screening strategies informed by risk-prediction models. Finally, the clinical outcomes and optimal strategies were sensitive to model-specific assumptions. Expanding comparative modeling analyses to additional simulation models, similar to USPSTF analyses, could provide more comprehensive insights and identify a common set of optimal or near-optimal strategies.

In conclusion, our study showed that stratifying FIT thresholds by age and sex may be cost-effective compared with current screening. The gain in expected health benefits with stratified FIT screening, however, is likely small.

## Supplementary Material

djaf149_Supplementary_Data

## Data Availability

Data underlying figures and tables will be shared upon request.
