# Supplementary material for: Benefits of colorectal cancer screening using fecal immunochemical testing with varying positivity thresholds by age and sex
Source: J Natl Cancer Inst. 2025 Jun 23;117(11):2219–28. doi: 10.1093/jnci/djaf149 (PMC12597507; doi:10.1093/jnci/djaf149)
Supplement: djaf149_Supplementary_Data [file djaf149_supplementary_data.zip › fit_psa_manuscript_supplementary_materials_clean.pdf]

Supplementary Material

Generating a model for FIT test characteristics by age, sex, and positivity threshold

We followed a multi-step process to estimate fecal immunochemical test (FIT) sensitivity and specificity for the detection of colorectal cancer (CRC), advanced adenomas (AA), and non-advanced adenomas (NAA). The microsimulation models used in this analysis require sensitivity values for adenomas of different sizes (≥ 10 mm, 6 to < 10 mm, 1 to <6 mm). We derived estimates for smaller adenoma sizes by applying scaling factors to the AA estimates. Specificity was defined as the probability of a negative test result in the absence of any neoplasia (CRC, AA, or NAA).

First, we used an adapted meta-analysis model for diagnostic accuracy [1] to estimate sensitivity for the detection of CRC. We fitted the model to age-, sex- and threshold-stratified data from a large cohort study using Markov chain Monte Carlo (MCMC) simulation. [2] This method models sensitivity and specificity simultaneously as a function of the positivity threshold and allows for interpolation and extrapolation beyond the thresholds reported in the study that inform the model. Moreover, for CRC, we treated each age-sex stratum as a separate study to allow for the borrowing of information between strata.

To derive test characteristics for the detection of AA, we conducted a separate meta-analysis using the same methodology to unstratified data from a previously published systematic review of FIT test characteristics. [3] We then calculated a scaling factor for each age- and sex stratum as the ratio of CRC sensitivity for an age-sex stratum over the combined pooled sensitivity for all strata. We applied the scaling factors to the unstratified AA test characteristics, assuming the relative differences in sensitivity and specificity by age and sex were equal to those for CRC.

To estimate the test characteristics for the detection of NAA, we calculated the ratio of NAA to AA sensitivity and specificity from a previous systematic review of FIT performance. [3] We then scaled the age- and sex-stratified test characteristics for AA from the previous step by this calculated ratio to derive stratified estimates for NAA. Additionally, we calculated separate sensitivities for small (6 to <10 mm) and diminutive (1 to <6 mm) NAAs based on the sensitivity for NAA. We also assumed the proportion of small to diminutive adenomas to be equal to that observed in the Dutch COCOS trial (81.4% of NAA were diminutive, and 18.6% were small).[4] Further, we assumed that diminutive adenomas do not bleed and have a sensitivity equal to the false positive rate. Finally, we used weighted averaging to calculate the sensitivity for small adenomas so that the combined sensitivity equaled the model-derived sensitivity for NAA.

Probabilistic Sensitivity Analysis

As we fitted separate meta-analysis models for CRC and AA, we sampled 100 parameter sets for the function relating threshold to sensitivity and specificity from two independent posterior distributions: one for CRC and one for AA. Therefore, the initial sensitivity estimates for AA may be higher than for CRC in some samples. To address this issue, we applied a rank-order algorithm developed by Goldhaber-Fiebert & Jalal [5] that induces a correlation between the sampled parameter values for CRC and AA by reordering the order of the samples without changing the marginal distributions. The sensitivities for NAA were derived from AA sensitivities using the same scaling factor as for the primary analysis. Finally, we performed 100 simulations with a population of 10 million individuals for each screening strategy.

Value of Information Analysis

Our probabilistic sensitivity analysis (PSA) enabled the calculation of expected loss curves (ELCs) to evaluate the economic impact of parameter uncertainty in FIT characteristics. ELCs visualize the expected opportunity costs of choosing suboptimal screening strategies across willingness-to-pay thresholds, providing insight into the value of reducing decision uncertainty. The height of an ELC at any willingness-to-pay threshold represents the expected value of perfect information (EVPI) - the maximum value to be gained by eliminating uncertainty in FIT performance characteristics.

To contextualize these findings at a population level, we calculated a cohort-specific lifetime EVPI by multiplying the per-person EVPI from our model (based on 1,000 40-year-olds) by the size of a single U.S. birth cohort reaching screening eligibility. Using 2020 U.S. resident population data [6] and published screening eligibility rates (83% of adults aged 45-49 years) [7], we estimated 1,981,694 males and 1,982,106 females would be eligible for screening upon reaching age 45. This cohort-specific projection captures the potential long-term value of reducing uncertainty in FIT performance for a single birth cohort across their lifetime, though it does not represent the full population-level impact across all screening-eligible age groups at any given time.

Supplemental Material References

1. Jones HE, Gatsonsis CA, Trikalinos TA, Welton NJ, Ades AE. Quantifying how diagnostic test accuracy depends on threshold in a meta-analysis. Stat Med. 2019;38(24):4789-803.
2. Selby K, Jensen CD, Lee JK, Doubeni CA, Schottinger JE, Zhao WK, et al. Influence of Varying Quantitative Fecal Immunochemical Test Positivity Thresholds on Colorectal Cancer Detection: A Community-Based Cohort Study. Ann Intern Med. 2018;169(7):439-47.
3. Lin JS, Perdue LA, Henrikson NB, Bean SI, Blasi PR. Screening for Colorectal Cancer: An Evidence Update for the US Preventive Services Task Force. U.S. Preventive Services Task Force Evidence Syntheses, formerly Systematic Evidence Reviews. Rockville (MD)2021.
4. Stoop EM, de Haan MC, de Wijkerslooth TR, Bossuyt PM, van Ballegooijen M, Nio CY, et al. Participation and yield of colonoscopy versus non-cathartic CT colonography in population-based screening for colorectal cancer: a randomised controlled trial. Lancet Oncol. 2012;13(1):55-64.
5. Goldhaber-Fiebert JD, Jalal HJ. Some Health States Are Better Than Others: Using Health State Rank Order to Improve Probabilistic Analyses. Med Decis Making 2016;36:927-40.
6. United States Census Bureau. Annual Estimates of the Resident Population by Single Year of Age and Sex for the United States: April 1, 2020 to July 1, 2023 (NC-EST2023-AGESEX-RES). https://www2.census.gov/programs-surveys/popest/datasets/2020-2023/national/asrh/nc-est2023-agesex-res.csv, 2024.
7. Piscitello A, Edwards DKt. Estimating the Screening-Eligible Population Size, Ages 45-74, at Average Risk to Develop Colorectal Cancer in the United States. Cancer Prev Res (Phila) 2020;13:443-448
8. Warren JL, Klabunde CN, Mariotto AB, Meekins A, Topor M, Brown ML, et al. Adverse events after outpatient colonoscopy in the Medicare population. Ann Intern Med. 2009;150(12):849-57, W152.
9. Gatto NM, Frucht H, Sundararajan V, Jacobson JS, Grann VR, Neugut AI. Risk of perforation after colonoscopy and sigmoidoscopy: a population-based study. J Natl Cancer Inst. 2003;95(3):230-6.
10. van Hees F, Zauber AG, Klabunde CN, Goede SL, Lansdorp-Vogelaar I, van Ballegooijen M. The appropriateness of more intensive colonoscopy screening than recommended in Medicare beneficiaries: a modeling study. JAMA Intern Med. 2014;174(10):1568-76.
11. Peterse EFP, Meester RGS, de Jonge L, et al. Comparing the Cost-Effectiveness of Innovative Colorectal Cancer Screening Tests. J Natl Cancer Inst 2021;113:154-161.

Supplementary Tables

Table S1. Evaluated screening strategies.

Screening Modality	Sex	Positivity Thresholds (µg/g)			Number of Unique Strategies
		Age range 1 (45-59 years)	Age range 2 (60-69 years)	Age range 3 (70-75 years)	
FIT (annual)	Female, Male	10, 20, 30, 40, 50	10, 20, 30, 40, 50	10, 20, 30, 40, 50	250

FIT, fecal immunochemical test

Table S2. Post-colonoscopy surveillance assumptions

Finding at second-most recent colonoscopy[a]	Finding at first-most recent colonoscopy[a]	Interval[b] to next colonoscopy, y
No prior colonoscopy	Normal colonoscopy[c]	See note below[d]
	1-2 adenomas <10 mm	7
	3-4 adenomas <10 mm	3
	10 adenomas <10 mm or any adenoma ≥10 mm	3
	> 10 adenomas	1
Normal colonoscopy[c]	Normal colonoscopy[c]	10
	1-2 adenomas <10 mm	7
	3-4 adenomas <10 mm	3
	5-10 adenomas <10 mm or any adenoma ≥10 mm	3
	> 10 adenomas	1
1-2 adenomas <10 mm	Normal colonoscopy[c]	10
	1-2 adenomas <10 mm	7
	3-4 adenomas <10 mm	3
	5-10 adenomas <10 mm or any adenoma ≥10 mm	3
	> 10 adenomas	1
3-4 adenomas <10 mm	Normal colonoscopy[c]	10
	1-2 adenomas <10 mm	7
	3-4 adenomas <10 mm	3
	5-10 adenomas <10 mm or any adenoma ≥10 mm	3
	> 10 adenomas	1
5-10 adenomas <10 mm or any adenoma ≥10 mm	Normal colonoscopy[c]	5
	1-2 adenomas <10 mm	5
	3-4 adenomas <10 mm	3
	5-10 adenomas <10 mm or any adenoma ≥10 mm	3
	> 10 adenomas	1
> 10 adenomas of any size	Normal colonoscopy[c]	5
	1-2 adenomas <10 mm	5
	3-4 adenomas <10 mm	3
	5-10 adenomas <10 mm or any adenoma ≥10 mm	3
	>10 adenomas	1

[a] This table omits the case where CRC is detected at a screening, diagnostic, or surveillance colonoscopy because MISCAN and SimCRC do not simulate detailed events following CRC diagnosis.

[b] The Multi-Society Task Force provides a range for some intervals (e.g., the interval for 3-4 adenomas <10 mm is 3-5 years). In such cases, we selected the shortest intervals provided.

[c] normal colonoscopy = no adenomas, SSPs (currently not simulated), or CRC is detected.

[d] A person whose first screening or diagnostic colonoscopy is normal does not enter surveillance but resumes screening 10 years after the normal colonoscopy.

Table S3. Colonoscopy test characteristics and complications assumptions

**Colonoscopy Test Characteristics**	
Sensitivity, %	
Small adenoma (< 6 mm)	75
Medium adenomas (6 to <10mm)	85
Large adenomas (≥ 10 mm)	95
CRC	95
Specificity, %	86
**Complication rates[a]**	
Fatal perforation	0.0000142391[b]
Serious gastrointestinal event[c]	Age-specific[f]
Other gastrointestinal event[d]	Age-specific[f]
Cardiovascular event[e]	Age-specific[f]

CRC = colorectal cancer; FIT = fecal immunochemical test.

[a] applies only for colonoscopies with polypectomy

[b] Risk of dying from a colonoscopy with polypectomy at age 65 (Warren et al. [8], Gatto et al. [9], and Van Hees et al. [10]).

[c] Serious gastrointestinal events are perforations, gastrointestinal bleeding, or transfusions.

[d] Other gastrointestinal events are paralytic ileus, nausea, and vomiting, dehydration, and abdominal pain.

[e] Cardiovascular events are myocardial infarction or angina, arrhythmias, congestive heart failure, cardiac or respiratory arrest, syncope, hypotension, or shock.

[f] Formulas for age-specific excess risks:

Serious GI events: $1 / [\exp(9.27953 - 0.06105 \times Age) + 1] - 1 / [\exp(10.78719 - 0.06105 \times Age) + 1]$

Other GI events: $1 / [\exp(8.81404 - 0.05903 \times Age) + 1] - 1 / [\exp(9.61197 - 0.05903 \times Age) + 1]$

Cardiovascular events: $1 / [\exp(9.09053 - 0.07056 \times Age) + 1] - 1 / [\exp(9.38297 - 0.07056 \times Age) +1]$

Table S4. Societal Costs of Screening, Follow-Up, Surveillance Tests, and Complications (2020 USD)

**Test costs**				
	Test costs ($)	Patient time (h)	Time costs ($)[a]	Total costs (societal, 2020 $)
Colonoscopy[b]				
Screening colonoscopy without lesion removal	891.37	39.93	805.39	1,696.76
Follow-up colonoscopy without lesion removal	891.01	39.93	805.39	1,696.40
Surveillance colonoscopy without lesion removal	890.65	39.93	805.39	1,696.04
Any colonoscopy with lesion removal	1,328.22	39.93	805.39	2,133.61
FIT[c]	23.82	1	20.17	43.99
**Complications**				
	Total complication cost (societal, 2020 $)			
Serious gastrointestinal event	13,573			
Other gastrointestinal event	8,574			
Cardiovascular event	10,985			

Abbreviations: FIT, fecal immunochemical test.
[a] We assumed an hour of patient time to equal the median wage rate in 2020 ($20.17) according to data from the US Bureau of Labor Statistics (BLS))
[b] Colonoscopy costs were based on an analysis of 2020 Centers for Medicare and Medicaid Services (CMS) data
[c] FIT costs were based on 2017 Clinical Laboratory Fee Schedule data, inflated to 2020 USD using 2020 CPI data for All Urban Consumers (CPI-U)

Table S5. Costs of Cancer Care (2020 USD)

**Phase of cancer care**	**Annual societal costs (2020 $)**[a]			
	Stage I	Stage II	Stage III	Stage IV
Initial phase	46,681	64,183	91,071	132,991
Continuing phase	4,695	5,397	8,139	37,134
Terminal phase, death CRC	90,080	100,829	105,300	130,973
Terminal phase, death other causes	27,782	29,471	38,235	84,111

Abbreviations: CRC, colorectal cancer
[a] from Peterse et al. [11], inflated to 2020 USD using 2020 CPI data for All Urban Consumers (CPI-U)

Table S6. Utility Losses of Screening, Follow-Up, Surveillance Tests, and Complications

**Utility loss for test procedure itself[a]**			
	Disutility	Time disutility is applied (h)	Utility loss per event
Colonoscopy	0.12	36.22	0.000496
FIT	0	1	0
**Utility loss for waiting for test results[a]**			
	Disutility	Time disutility is applied (h)	Utility loss per event
Colonoscopy without lesion removal	0	0	0
Colonoscopy with lesion removal	0.033036	10	0.000905
FIT	0.003304	7	0.000063
**Utility loss for waiting for diagnostic follow-up colonoscopy[a]**			
	Disutility	Time disutility is applied (h)	Utility loss per event
FIT	0.033036	14	0.001267
**Total utility loss per test[a]**			
	Disutility negative test	Disutility positive test	
Colonoscopy	0.000496	0.001401	
FIT	0.000063	0.001330	
**Utility loss for complications[a]**			
	Disutility	Time disutility is applied (h)	Utility loss per event
Serious gastrointestinal event	0.5	96	0.0055
Other gastrointestinal event	0.5	48	0.0027
Cardiovascular event	0.5	84	0.0048

Abbreviations: fecal immunochemical test.
[a] from Peterse et al. [11]

Table S7. Utility Losses of Cancer Care

**Phase of cancer care**	**Utility loss[a]**			
	Stage I	Stage II	Stage III	Stage IV
Initial phase	0.12	0.18	0.24	0.70
Continuing phase	0.05	0.05	0.24	0.70
Terminal phase, death CRC	0.70	0.70	0.70	0.70
Terminal phase, death other causes	0.05	0.05	0.24	0.70

[a] from Peterse et al. [11]

Supplementary Figures

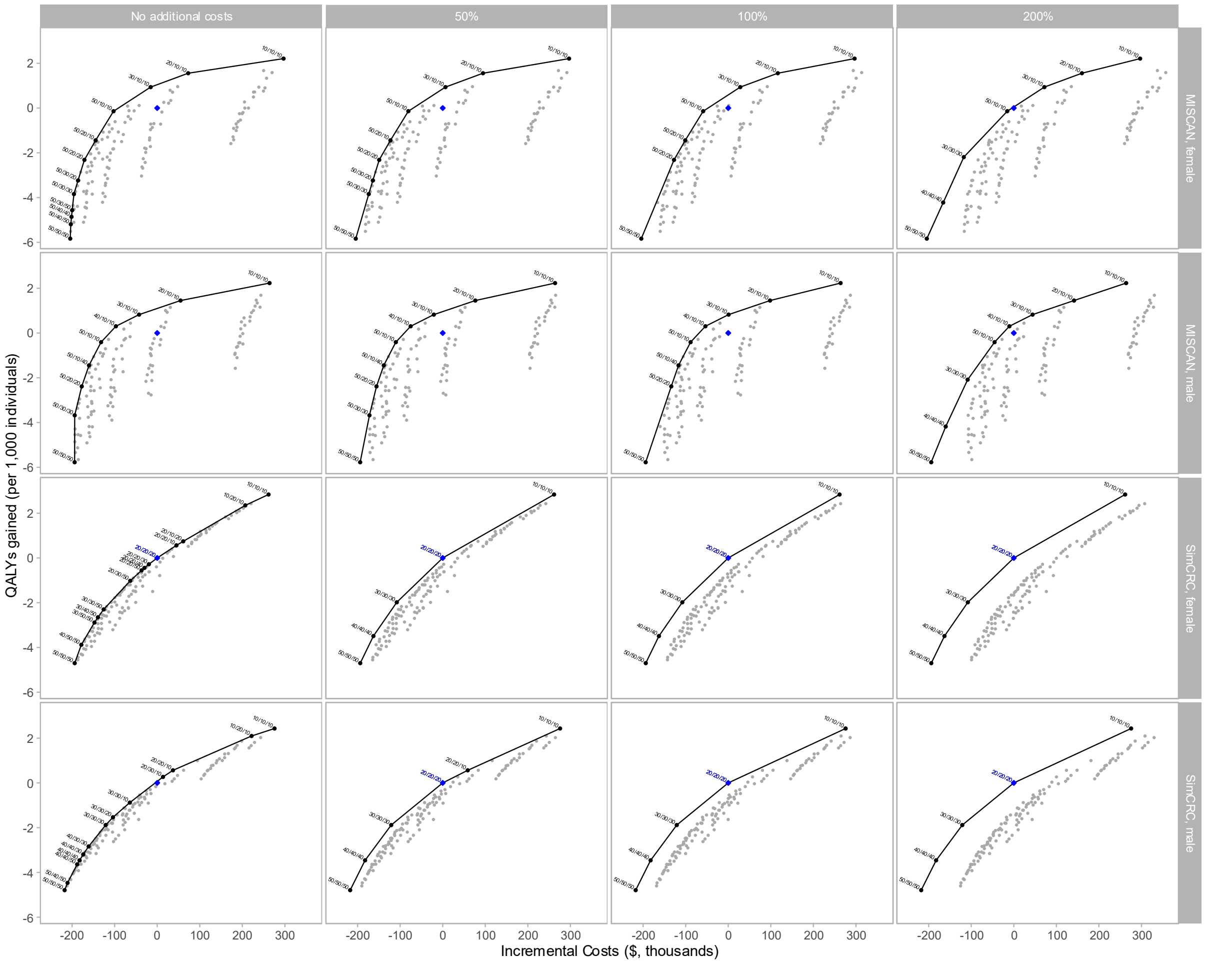

Figure S1. Costs and quality-adjusted life-years gained for all FIT threshold combinations by model and sex, with increased costs for non-uniform thresholds (additional costs of 50%, 100%, and 200% of a single FIT). Strategies are labeled 'X/Y/Z', where X, Y, and Z represent thresholds in µg/g for ages 45-59, 60-70, and 70-75, respectively.

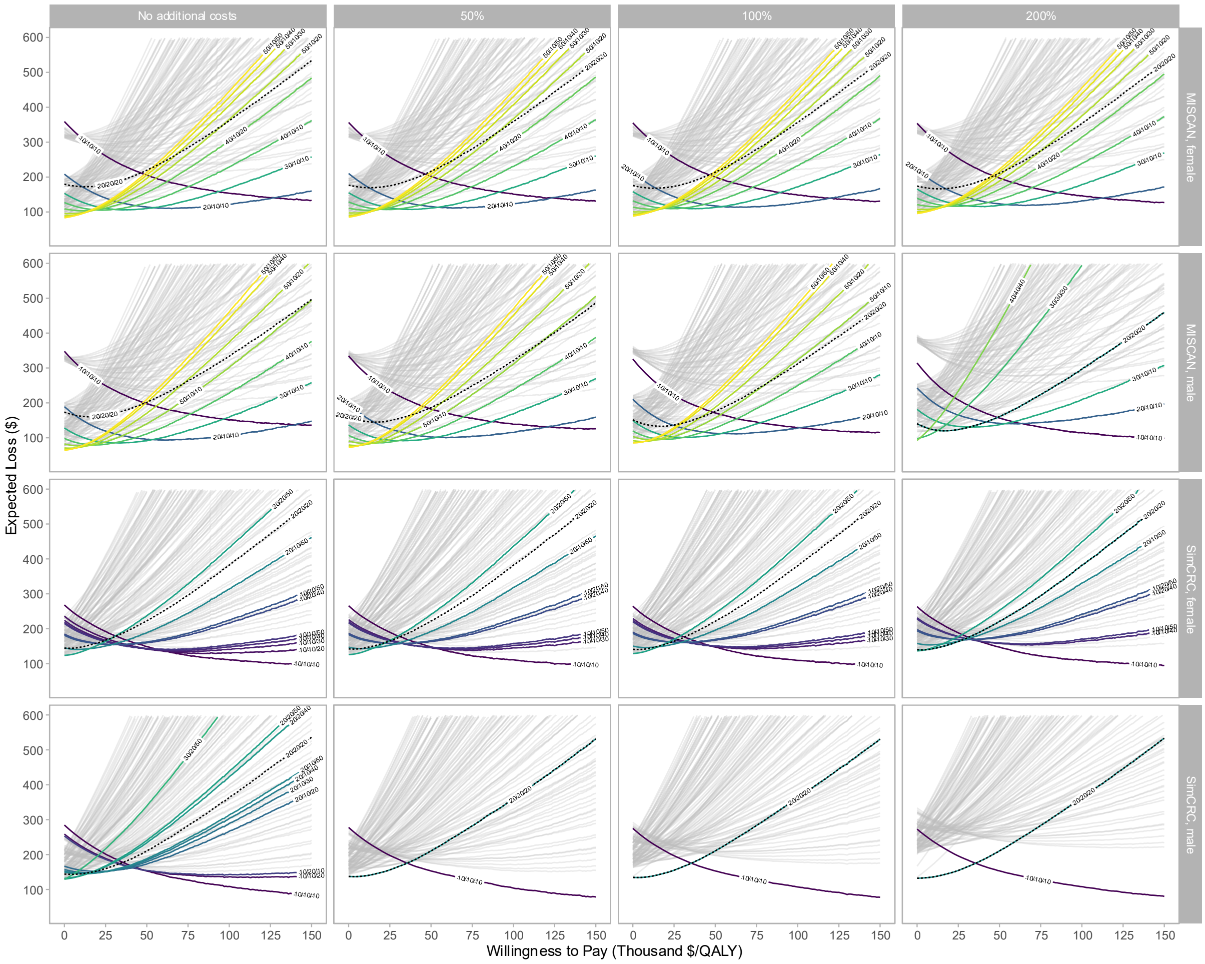

Figure S2. Expected loss per individual for all FIT threshold combinations with added one-time implementation costs at age 45 for non-uniform thresholds (additional costs of 50%, 100%, and 200% of a single FIT). Strategies are labeled 'X/Y/Z', where X, Y, and Z represent thresholds in µg/g for ages 45-59, 60-70, and 70-75, respectively. The colored lines represent strategies that minimize the expected loss for some value of the willingness-to-pay threshold. The black dotted line represents the current uniform threshold of 20µg/g.

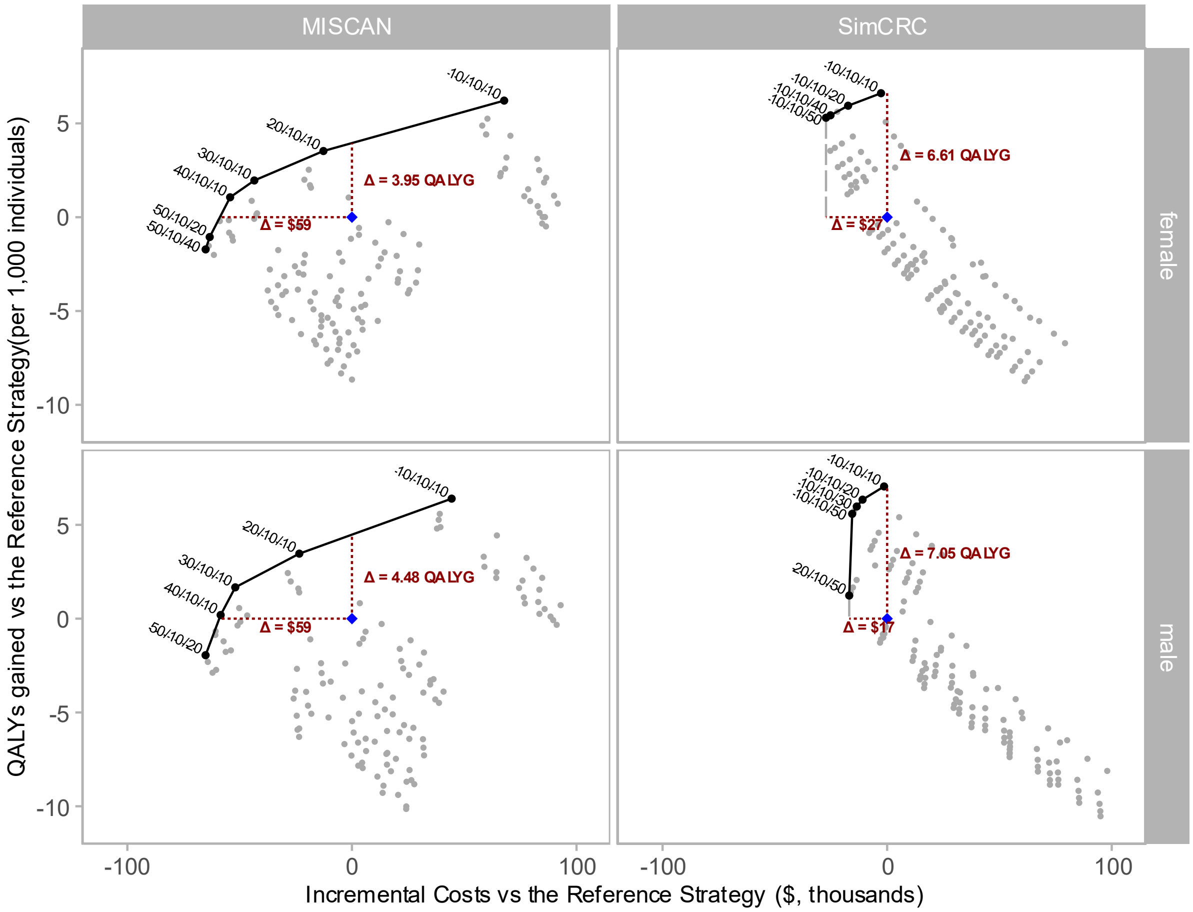

Figure S3. Costs and quality-adjusted life-years gained for all FIT threshold combinations by model and sex, assuming 60% adherence to FIT screening, diagnostic follow-up colonoscopies and colonoscopy surveillance. Strategies are labeled 'X/Y/Z', where X, Y, and Z represent thresholds in µg/g for ages 45-59, 60-70, and 70-75, respectively. The blue point marks the current uniform FIT threshold of 20µg/g, which is the reference strategy.

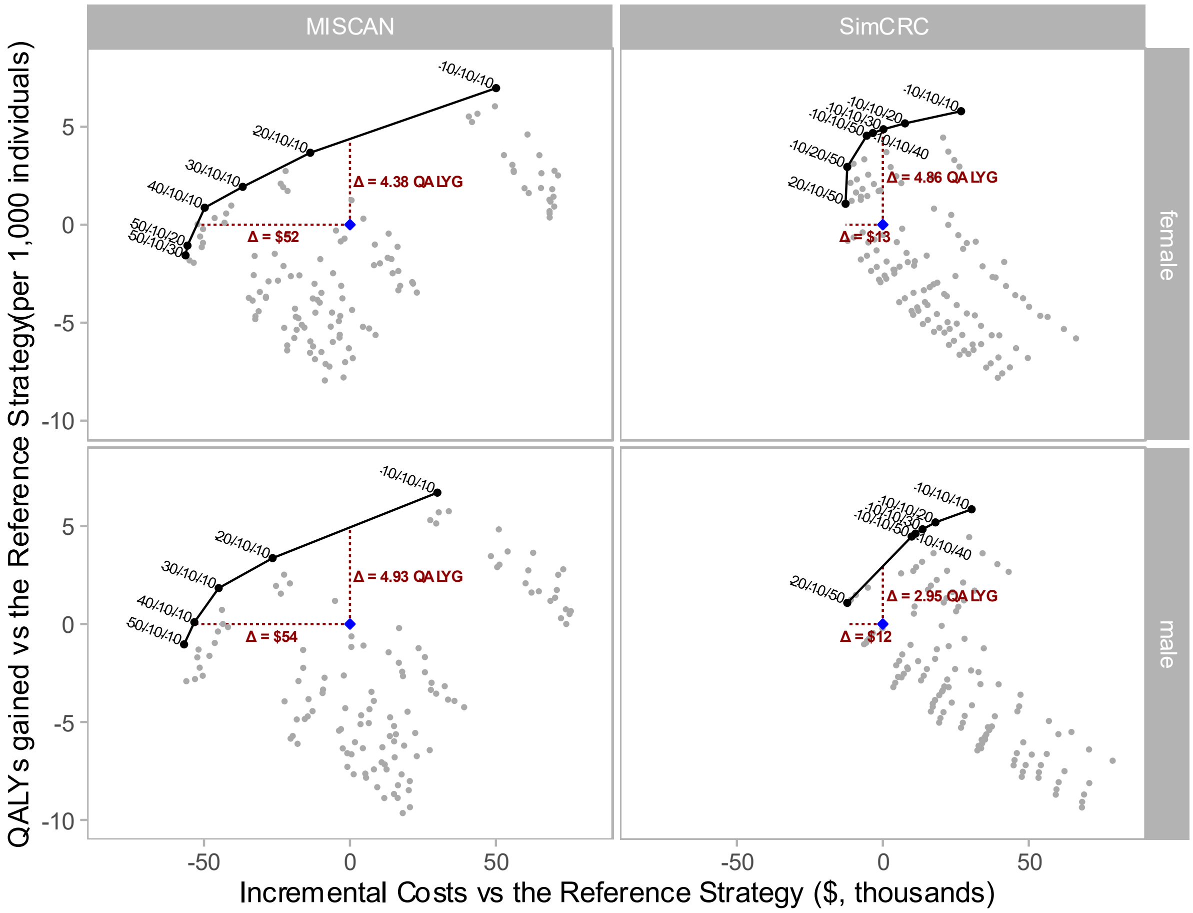

Figure S4. Costs and quality-adjusted life-years gained for all FIT threshold combinations by model and sex, assuming imperfect adherence to FIT screening, diagnostic follow-up colonoscopies and colonoscopy surveillance. We assumed 10% of individuals would never attend screening and 30% would always participate. The remaining population has a 60% probability of attending the first screening test. After the initial test, those who attended have a 75% chance of attending the next screening, while non-attenders have a 25% chance of attending. Strategies are labeled 'X/Y/Z', where X, Y, and Z represent thresholds in µg/g for ages 45-59, 60-70, and 70-75, respectively. The blue point marks the current uniform FIT threshold of 20µg/g, which is the reference strategy.

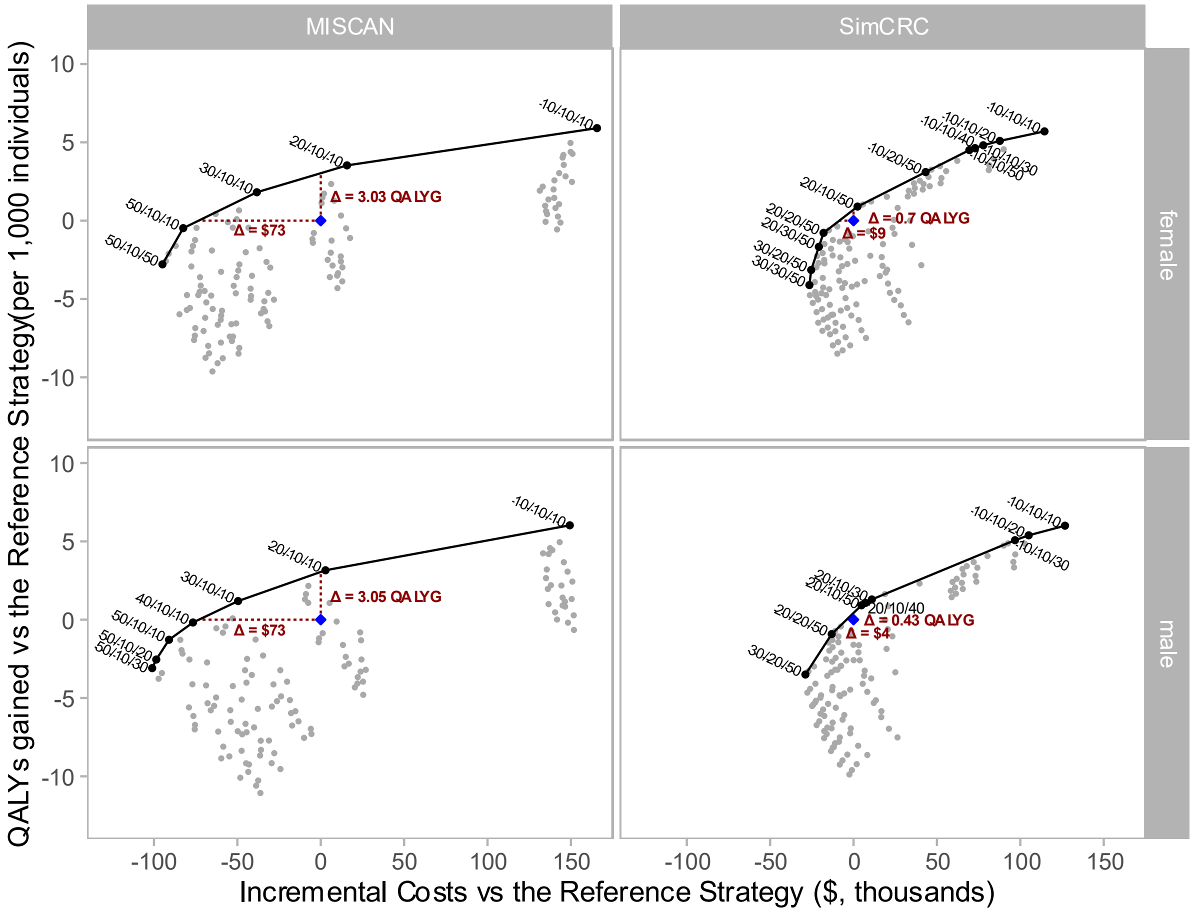

Figure S5. Costs and quality-adjusted life-years gained for all FIT threshold combinations by model and sex, assuming biennial FIT screening intervals. Strategies are labeled 'X/Y/Z', where X, Y, and Z represent thresholds in µg/g for ages 45-59, 60-70, and 70-75, respectively. The blue point marks the current uniform FIT threshold of 20µg/g, which is the reference strategy.

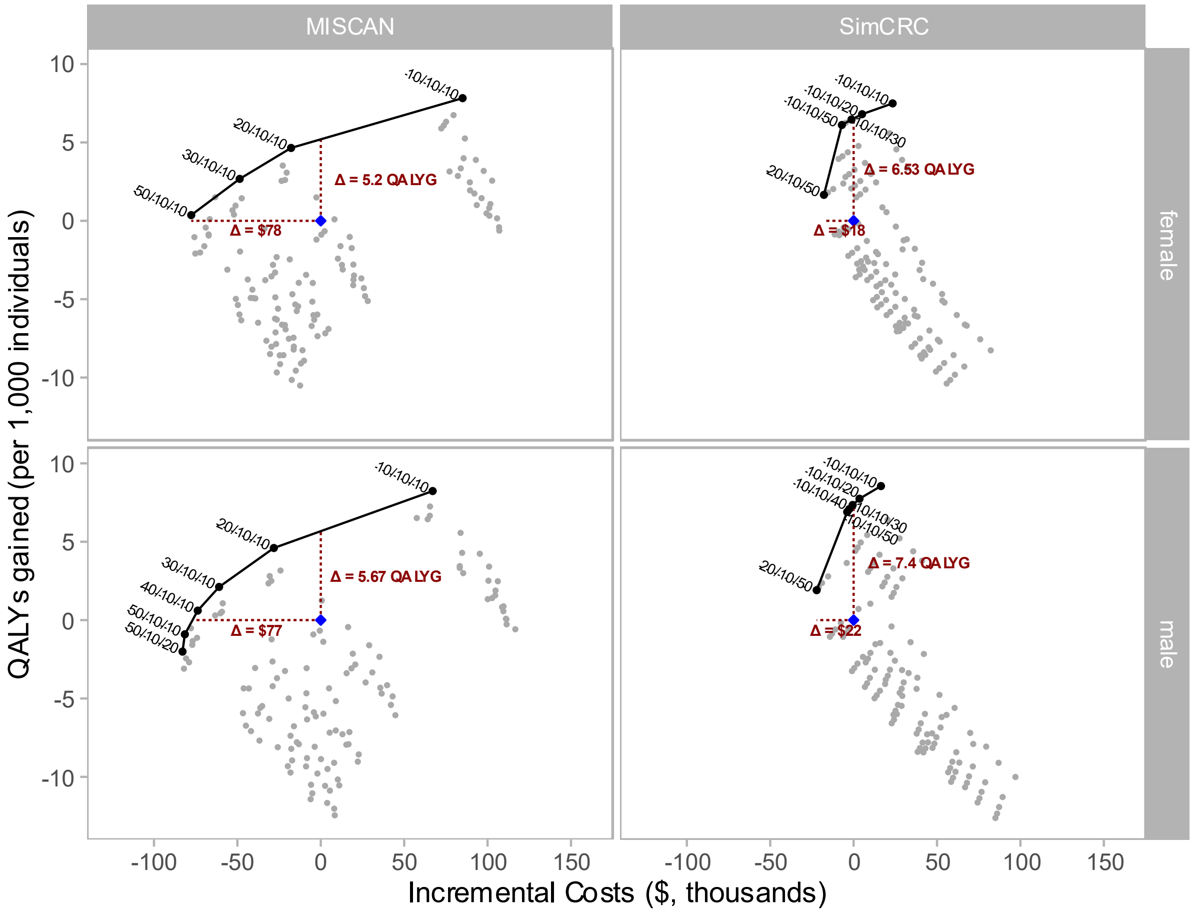

Figure S6. Costs and quality-adjusted life-years gained for all FIT threshold combinations by model and sex, assuming triennial FIT screening intervals. Strategies are labeled 'X/Y/Z', where X, Y, and Z represent thresholds in µg/g for ages 45-59, 60-70, and 70-75, respectively. The blue point marks the current uniform FIT threshold of 20µg/g, which is the reference
